# Epistatic interactions of major effect drought QTLs with genetic background loci determine grain yield of rice under drought stress

**DOI:** 10.1038/s41598-019-39084-7

**Published:** 2019-02-22

**Authors:** Shailesh Yadav, Nitika Sandhu, Ratna Rani Majumder, Shalabh Dixit, Santosh Kumar, S. P. Singh, N. P. Mandal, S. P. Das, Ram Baran Yadaw, Vikas Kumar Singh, Pallavi Sinha, Rajeev K. Varshney, Arvind Kumar

**Affiliations:** 1 Rice Breeding Platform, International Rice Research Institute, DAPO Box 7777 Metro Manila, Philippines; 2 ICAR Research Complex for Eastern Region, Patna, Bihar India; 3 Bihar Agricultural University, Sabour, Bihar India; 4 Central Rainfed Upland Rice Research station, National Rice Research Institute, Hazaribagh, Jharkhand India; 5 ICAR Research Complex for NEH Region, Tripura Centre, Lembucherra, Tripura India; 6 Regional Agriculture Research Station, NARC, Bara, Nepal; 7 International Rice Research Institute, South Asia Hub, ICRISAT, Patancheru, Hyderabad India; 8 International Crops Research Institute for the Semi-arid Tropics, Patancheru, Hyderabad India

## Abstract

Epistatic interactions of QTLs with the genetic background and QTL-QTL interaction plays an important role in the phenotypic performance of introgression lines developed through genomic-assisted breeding (GAB). In this context, NIL pairs developed with various drought QTL (*qDTY*) combinations in the genetic background of IR64, TDK1-*Sub1* and Savitri backgrounds were utilized to study the interactions. Multi-season phenotyping of NIL pairs harboring similar *qDTY* combinations provided contrasting performance for grain yield under drought (RS) (classified as high and low yielding NILs) but nearly similar performance under non-stress(NS) conditions. Genome wide genotyping data revealed a total of 16, 5 and 6 digenic interactions were detected under RS conditions in low yielding NILs of IR64, TDK1-*Sub1* and Savitri respectively while no significant interaction was found in high yielding NILs under RS and NS conditions in any of the genetic backgrounds used in this study. It is evident from this study that existence of epistatic interactions between QTLs with genetic background, QTL-QTL interaction and interactions among background markers loci itself on different chromosomes influences the expression of a complex trait such as grain yield under drought. The generated information will be useful in all the GAB program of across the crops for precise breeding.

## Introduction

Drought is one of the most severe climatic threat to rainfed rice production affecting on more than 23 million hectares of South and Southeast Asia^1^. A further increase in drought intensity has anticipated due to ongoing global climatic changes. Development of climate-adapted crop varieties is an urgent need to counter the effect of more than 50% yield losses due to abiotic stresses^2,3^. Widely grown high yielding rice varieties are highly sensitive to drought. The recent advances in molecular marker and genomics technologies have played a major role in selection of drought-tolerant traits. Rice drought breeding programme at IRRI has successfully identified and validated some of the major and consistent effect grain yield QTLs under reproductive stage drought (*qDTYs*) such as *qDTY*_*1*.*1*_^4,5^, *qDTY*_*2*.*1*_^6^, *qDTY*_*2*.*2*_^7^, *qDTY*_*3*.*1*_^6^, *qDTY*_*3*.*2*_^4^, *qDTY*_*6*.*1*_^8^ and *qDTY*_*12*.*1*_^9^ using molecular markers. A considerable effort has been made to improve drought susceptible mega varieties of rice through marker-assisted introgression of *qDTYs*^10–13^. Assembling multiple alleles/QTLs/genes in a genetic background using marker-assisted introgression may be an appropriate approach to achieve the expected phenotypic variance in enhancing rice grain yield under drought stress^7,12,14^, submergence tolerance^15^, salt tolerance^16^, cold tolerance^17^, resistance to blast^18–20^, bacterial blight^21–23^, brown planthopper^24,25^. The introgression of major effect QTLs/genes and its combinations do not always result in desirable improvement for the targeted trait(s). In some cases, the effect of identified genetic loci in a QTL/gene pyramiding program is not sufficient to fully explain the desired phenotypic variation due to various interactions occurring for a complex quantitative trait. The possible interactions between the genetic make-up, epistasis, pleiotropy and linkage among/between the introgressed loci, with the genetic background and environment could have negative/positive effects on the expression of the traits that should never be underestimated^26–28^. QTL pyramiding approach has enormous potential to improve our understanding of interactions among QTLs and also help to develop new strategies to increase the efficiency of marker-assisted selection program. In this context, development of NILs using marker assisted selection (MAS) is a way forward to verify the effects of one or few major effect QTLs as well as increase the recovery of recurrent parent genome at a faster rate^29^. Rice NILs for complex traits such as drought^7,13^, blast^19,30^ and brown planthopper^25,31^ were developed and evaluated in different genetic backgrounds of popular rice varieties.

NILs harboring QTLs/genes for abiotic/biotic stresses in the elite backgrounds with superior performances are useful material for immediate release to farmer’s field. NILs are also useful source to study the stable epistatic interactions between the introgressed donor segment and the rest of the recurrent parent genome which gives an important clue about differences in performance of the same set of NILs under variable conditions. The expressions of QTLs/genes for complex traits are strongly affected by the genetic backgrounds^32,33^. The estimation of genetic backgrounds or recurrent parent genome (RPG) recovery with different types of markers and number of markers also affect the estimation of RPG recovery as a good number of markers with good coverage estimates better information^19^. Better estimation of RPG recovery at early generation of selection can be useful to eliminate linkage drags which can cause unexpected traits in the MAS breeding products because of neighboring genes of the target QTLs. Also, the favorable allele at one locus in a particular background may become unfavorable in another genetic background due to the epistatic interaction^34^. Role of epistasis phenomena in plant breeding has been discussed for a long time, but the extent of the expression level of quantitative traits is due to this complex genetic phenomena are poorly understood^35^. Epistatic or non-allelic interaction is a genetic factor and may change the magnitude of phenotypic expression of QTLs by suppressing/enhancing the loci effect associated with a trait to the other loci of genetic background^34,36^. The epistatic effect is a deviation from the sum of independent effects of QTLs/genes therefore biased estimation of QTL effects is possible in genetic models assuming no interaction in QTL mapping studies^37,38^. The epistatic interaction of QTLs and background or modifying loci has been recently reported for quantitative traits in various crops^38–41^.

Genomic interactions play an important role in deciding the performance of MAS lines for grain yield under drought and various significant QTL x QTL interactions in pyramided lines have been reported for drought-related traits^12,28,42,43^. Most of these studies highlighted capture of such interactions for increased performance of QTLs pyramided lines under drought. But none of these studies reported reasons for a lower yield of NILs with same combinations of pyramided QTLs in the same or different recipient backgrounds. Keeping in this view, the present study is an effort (i) to evaluate the performance of drought NILs of IR64, TDK1-*Sub1* and Savitri backgrounds under reproductive stage moderate and severe drought stress and non-stress conditions (ii) to identify promising drought tolerant NILs with higher recovery of recipient genome (iii) to study epistatic interaction of major effect grain yield drought QTLs and with background marker loci.

## Results

### Phenotypic performance of drought NILs possessing *qDTY* under RS and NS conditions

Significant phenotypic differences under RS and NS were observed among the parents and NILs in IR64, TDK1-*Sub1* and Savitri backgrounds. The results of ANOVA, means, LSD, heritability of IR64 NILs with a *qDTY* and *qDTY* combinations under RS (SS and MS) and NS are summarized in Table 1. Among IR64 NILs, the average yield reduction was found 87% under SS and 74% under MS as compared to NS conditions. Trial mean of NILs in IR64 background for GY was 690 kg ha^−1^ under SS in 2015 DS while it was 1358 kg ha^−1^ under MS in 2017 DS. Broad-sense heritability (H) of GY under RS ranged from 0.78 to 0.89 in the years of 2015 and 2017 DS. The NIL pairs harboring same *qDTY or qDTY* combinations with on par background recovery, provided significantly different grain yield (GY) under similar level of drought stress were classified into high and low yielding NILs (Table 1). For instance, nine IR64 NILs possessing *qDTY*_*12*.*1*_ QTL yielded 25 to 3375 kg ha^−1^ under SS situation, whereas no such difference was observed under NS situation (4490 to 6470 kg ha^−1^). Another four IR64 NILs possessing *qDTY*_*2*.*3*_ yielded 480 kg ha^−1^ to 2495 kg ha^−1^ under MS. In NS conditions, trial mean for GY was 5360 kg ha^−1^ and ranged from 4375 kg ha^−1^ to 6958 kg ha^−1^. Nine NILs with same combination of *qDTY*_*1*.*2*_ + *qDTY*_*12*.*1*_ have shown a large variation for grain yield under drought and ranged from 89 to 2141 kg ha^−1^. The huge variation in grain yield under drought even having same QTL combinations was might be due to some hidden interactions and analysis of such interaction were explored and discussed in this study. Similarly three NILs with *qDTY*_*2*.*2*_ + *qDTY*_*2*.*3*_ yielded 572 to 1455 kg ha^−1^; four NILs with *qDTY*_*4*.*1*_ + *qDTY*_*12*.*1*_ yielded 369 to 936 kg ha^−1^, two NILs with *qDTY*_*1*.*1*_ + *qDTY*_*1*.*2*_ + *qDTY*_*12*.*1*_ yielded 289 to 611 kg ha^−1^ and two NILs with *qDTY*_*4*.*1*_ yielded 414 to 1031 kg ha^−1^_._ The mean plant height (PH) of IR64 NILs under RS was higher than the recurrent background IR64 while PH of NILs under NS was very similar to that of IR64 (Table 1). Days to flowering (DTF) were affected significantly with severity of stress and flowering delay of 10 days under SS and 7 days under MS was observed.

**Table 1:** Mean performance for grain yield (GY), days to flowering (DTF) and plant height (PH) of IR64 NILs under severe stress (SS), moderate stress (MS) and non-stress (NS) conditions.

NILs	QTL and QTLs combinations	GY (kg ha^−1^)			DTF (days)			PH (cm)			Classification
		2015DS	2017DS	2017DS	2015DS	2017DS	2017DS	2015DS	2017DS	2017DS	
		SS	MS	NS	SS	MS	NS	SS	MS	NS	
IR 102793:1-11-66-3-1-1	*qDTY*_*1*.*2*_ + *qDTY*_*12*.*1*_	2141	1455	4375	74	73	71	75	67	91	High yielding
IR 102796-14-140-1-1-1	*qDTY*_*1*.*2*_ + *qDTY*_*12*.*1*_	788	2740	4928	85	79	78	64	66	92	High yielding
IR 102793:1-11-192-1-1-4	*qDTY*_*1*.*2*_ + *qDTY*_*12*.*1*_	1386	1110	4748	84	82	79	64	64	89	High yielding
IR 102793:1-11-64-1-1-2	*qDTY*_*1*.*2*_ + *qDTY*_*12*.*1*_	2002	2750	6145	75	75	76	67	66	90	High yielding
IR 102784:2-42-17-2-1-1	*qDTY*_*1*.*2*_ + *qDTY*_*12*.*1*_	540	1810	6191	92	83	96	55	87	92	High yielding
IR 102796-14-132-1-1-3	*qDTY*_*1*.*2*_ + *qDTY*_*12*.*1*_	89	515	5855	85	84	81	55	58	86	Low yielding
IR 102796-14-65-2-1-1	*qDTY*_*1*.*2*_ + *qDTY*_*12*.*1*_	138	1050	5990	86	77	76	58	60	92	Low yielding
IR 102784:2-42-16-1-1-2	*qDTY*_*2*.*3*_ + *qDTY*_*3*.*2*_	1426	1860	5430	78	72	70	60	71	92	High yielding
IR 102784:2-62-481-2-1-1	*qDTY*_*2*.*3*_ + *qDTY*_*3*.*2*_	191	1255	5200	95	84	82	59	78	92	Low yielding
IR 102783:2-70-112-2-1-4	*qDTY*_*12*. *1*_	1580	1640	4615	84	80	75	63	64	93	High Yielding
IR 102783:2-70-112-3-1-1	*qDTY*_*12*. *1*_	3375	2210	5143	74	75	71	75	68	96	High yielding
IR 102784:2-42-88-2-1-2	*qDTY*_*12*. *1*_	1675	2280	6390	83	77	75	62	62	92	High yielding
IR 102783:2-70-135-4-1-1	*qDTY*_*12*. *1*_	996	1225	4561	77	76	71	61	56	80	High yielding
IR 102783:2-70-139-2-1-2	*qDTY*_*12*. *1*_	1194	2060	5848	77	76	73	67	63	97	High yielding
IR 102784:2-42-99-2-1-3	*qDTY*_*12*. *1*_	209	1330	4820	96	83	79	55	63	92	Low yielding
IR 102784:2-90-385-1-1-3	*qDTY*_*12*. *1*_	160	1485	6470	94	79	78	54	66	87	Low yielding
IR 102784:2-62-434-1-1-1	*qDTY*_*12*. *1*_	25	645	4490	99	84	83	55	61	89	Low yielding
IR 102784:2-90-385-3-1-1	*qDTY*_*12*. *1*_	39	810	4780	96	83	81	52	51	93	Low yielding
IR 102784:2-62-66-1-1-2	*qDTY*_*1*.*1*_ + *qDTY*_*1*.*2*_ + *qDTY*_*12*.*1*_	611	845	5255	88	81	79	100	87	115	High yielding
IR 102784:2-118-22-1-1-2	*qDTY*_*1*.*1*_ + *qDTY*_*1*.*2*_ + *qDTY*_*12*.*1*_	289	1595	6068	91	80	80	55	61	86	Low yielding
IR 102784:2-42-3-1-1-2	*qDTY*_*2*.*2*_ + *qDTY*_*2*.*3*_	1455	1960	5465	75	73	70	64	78	84	High yielding
IR 102796-14-124-1-1-3	*qDTY*_*2*.*2*_ + *qDTY*_*2*.*3*_	996	2235	5220	85	73	69	62	87	91	High yielding
IR 102784:2-42-136-1-1-3	*qDTY*_*2*.*2*_ + *qDTY*_*2*.*3*_	572	1255	4988	87	75	70	57	78	81	Low yielding
IR 102784:2-42-127-3-1-1	*qDTY*_*2*. *3*_	1692	730	4497	94	75	73	58	82	84	High yielding
IR 102793:1-11-69-1-1-3	*qDTY*_*2*. *3*_	1667	2495	5600	74	73	71	61	80	88	High yielding
IR 102784:2-42-136-2-1-1	*qDTY*_*2*. *3*_	33	480	6865	103	84	78	49	82	85	Low yielding
IR 102784:2-42-138-1-1-1	*qDTY*_*2*. *3*_	157	1650	6270	98	75	75	59	83	85	Low yielding
IR 102793:1-11-189-2-1-2	*qDTY*_*4*. *1*_	1031	1720	6290	89	83	73	63	91	98	High yielding
IR 102784:2-118-15-1-1-2	*qDTY*_*4*. *1*_	414	1625	6958	92	82	80	57	84	91	Low yielding
IR 102784:2-118-549-2-1-2	*qDTY*_*4*.*1*_ + *qDTY*_*12*.*1*_	852	2125	6378	88	82	79	57	84	90	High yielding
IR 102793:1-11-229-3-1-1	*qDTY*_*4*.*1*_ + *qDTY*_*12*.*1*_	656	1900	5618	87	81	78	59	83	90	High yielding
IR 102784:2-89-284-3-1-1	*qDTY*_*4*.*1*_ + *qDTY*_*12*.*1*_	936	1175	5502	87	83	80	59	83	90	High yielding
IR 102784:2-89-284-1-1-3	*qDTY*_*4*.*1*_ + *qDTY*_*12*.*1*_	369	1600	6145	97	83	82	52	82	90	Low yielding
IR64	—	642	715	5910	88	79	78	63	65	93	Background
IR 86918-B-315	*qDTY*_*1*.*1*_, *qDTY*_*1*.*2*_	654	800	4715	78	88	84	101	88	107	Donor
Way Rarem	*qDTY*_*12*. *1*_	271	510	5050	98	80	96	86	80	122	Donor
IR 77298-14-1-2-17	*qDTY*_*2*.*2*_, *qDTY*_*4*.*1*_	490	1930	3868	87	82	81	93	79	99	Donor
Vandana	*qDTY*_*2*.*3*_, *qDTY*_*2*.*3*_	2074	1890	4760	75	73	72	89	76	92	Donor
Trial Mean		690	1358	5360	90	83	80	69	82	93	
LSD_0.05_		848.44	998	1416	9.52	8.52	5.9	10.92	8.92	10.16	
H		0.78	0.89	0.58	0.82	0.85	0.92	0.91	0.80	0.88	

Note: LSD_0.05_: least significant difference at the 5% confidence level; H: Broad sense heritability.

Mean performances of measured traits of TDK1-*Sub1* NILs, heritability and LSD is presented in Table 2. Grain yield under severe stress (GYSS) ranged from 0 kg ha^−1^ to 479 kg ha^−1^ with trial mean of 327 kg ha^−1^. The recurrent parent TDK1-*Sub1* yielded 14.4 kg ha^−1^ while the donor parent IR55419-04 performed well with GY of 881 kg ha^−1^ under SS situation. GY mean of MS trial (GYMS) was 889 kg ha^−1^, with range of 300 kg ha^−1^ to 1555 kg ha^−1^. The average yield reduction was more than 90% under SS while 84% less grain yield was recorded under MS among TDK1-*Sub1* NILs. Heritability for GY was recorded 0.89 to 0.91 in RS and 0.58 in NS. The pyramided three NILs with *qDTY*_*6*.*1*_ + *qDTY*_*6*.*2*_ combination yielded 0 to 479 kg ha^−1^ under SS situation. However, the performance of both lines was 300 and 1555 kg ha^−1^ respectively in MS. Similarly, NIL pair consisting of *qDTY*_*3*.*1*_ + *qDTY*_*6*.*1*_ + *qDTY*_*6*.*2*_ combination yielded 86 kg ha^−1^ and 479 kg ha^−1^ under SS and 1085 kg ha^−1^ to 1555 kg ha^−1^ under MS. A reduction in PH was observed under SS and MS conditions compared to NS condition. The mean PH under SS, MS and NS were 64 cm, 69 cm and 92 cm, respectively. Significant differences in DTF with trial mean of 94 days and flowering differences of 7 days between entries was observed under RS. Earliest line flowered in 80 days while most of the lines were of late maturing type and it flowered in 96 days. As observed in the case of IR64 NILs, TDK1-*Sub1* NILs was also produced nearly similar grain yield under NS situation.

**Table 2:** Mean performances for grain yield (GY), days to flowering (DTF) and plant height (PH) of TDK-*Sub1* and Savitri NILs under severe stress (SS), moderate stress (MS) and non- stress (NS).

NILS	QTL and QTLs combinations	GY (kg ha^−1^)			DTF (days)			PH (cm)			Background	Classification
		2015DS	2017DS	2017DS	2015DS	2017DS	2017DS	2015DS	2017DS	2017DS		
		SS	MS	NS	SS	MS	NS	SS	MS	NS		
IR 102777-5-83-1-2-7	*qDTY*_*6*.*1*_ + *qDTY*_*6*.*2*_	316	465	5590	95	78	78	93	96	126	TDK-*Sub1*	High yielding
IR 102774-26-8-3-2-5	*qDTY*_*6*.*1*_ + *qDTY*_*6*.*2*_	0	300	4915	96	99	85	66	65	108	TDK-*Sub1*	Low yielding
IR 102777-6-86-2-2-7	*qDTY*_*6*.*1*_ + *qDTY*_*6*.*2*_	163	540	5695	89	83	84	67	60	104	TDK-*Sub1*	Low yielding
IR 102777-6-86-2-2-11	*qDTY*_*3*.*1*_ + *qDTY*_*6*.*1*_ + *qDTY*_*6*.*2*_	479	1555	4966	80	77	74	71	66	93	TDK-*Sub1*	High yielding
IR 102774-15-32-3-1-2	*qDTY*_*3*.*1*_ + *qDTY*_*6*.*1*_ + *qDTY*_*6*.*2*_	86	1085	4958	84	76	76	93	84	129	TDK-*Sub1*	Low yielding
TDK1-*Sub1*	—	14.4	527	5412	70	80	82	61	71	110	Recipient	—
IR55419–04	*qDTY*_*3*.*1*_, *qDTY*_*6*.*1*_, *qDTY*_*6*.*2*_	881.5	652	5361	72	89	102	68	70	92	Donor	—
Trial Mean		327	889	5847	94	93	88	64	69	92		
LSD_0.05_		305	929	1371	6.8	2.7	1.15	17.8	5.08	12.8		
H		0.91	0.89	0.58	0.93	0.85	0.92	0.89	0.80	0.88		
IR 106523-21-28-1-2-B	*qDTY*_*3*. *2*_	517	2565	7098	72	78	78	68	61	90	Savitri	High yielding
IR 106531-4-22-3-3-B	*qDTY*_*3*. *2*_	134	2185	6478	78	82	78	74	68	90	Savitri	Low yielding
IR 106522-41-8-3-B	*qDTY*_*3*.*2*_ + *qDTY*_*12*.*1*_	627	1650	6035	72	76	74	76	65	92	Savitri	High yielding
IR 106523-3-9-3-2-B	*qDTY*_*3*.*2*_ + *qDTY*_*12*.*1*_	545	2165	6058	75	77	81	63	58	79	Savitri	High yielding
IR 106531-13-31-2-3-B	*qDTY*_*3*.*2*_ + *qDTY*_*12*.*1*_	145	1425	5725	76	78	77	61	60	78	Savitri	Low yielding
IR 106529-15-33-1-2-B	*qDTY*_*3*.*2*_ + *qDTY*_*12*.*1*_	18	1090	5035	81	80	79	85	75	102	Savitri	Low yielding
Savitri	—	48	903	6641	113	96	103	48	66	104	Recipient	—
IR 77298-5-6-18	*qDTY*_*3*. *2*_	236	1767	6393	96	84	88	57	57	84	Donor	—
IR74371-46-1-1	*qDTY*_*12*. *1*_	999	1715	4238	76	73	70	74	73	98	Donor	
Trial Mean		563	2319	6069	88	80	83	61	64	87		
LSD_0.05_		540	522	808	8.8	3.21	2.12	6.46	4.33	7.07		
H		0.74	0.89	0.58	0.84	0.85	0.92	0.72	0.80	0.88		

Note: LSD_0.05_: least significant difference at the 5% confidence level; H: Broad sense heritability.

The same traits were also observed in the Savitri NILs (Table 2). A reduction in mean GY under SS and MS compared with mean GY under NS was 90% and 61%, respectively. The recurrent parent Savitri yielded 48 kg ha^−1^ while the donor parent IR77298-5-6-18 yielded 236 kg ha^−1^ under SS situation. NIL pair carrying single *qDTY*_*3*.*2*_ yielded 134 to 517 kg ha^−1^ under SS, whereas four NILs carrying two *qDTY* (*qDTY*_*3*.*2*_ + *qDTY*_*12*.*1*_) yielded 18 to 627 kg ha^−1^. The significant deviation observed in GY suggesting some negative interaction between background markers loci.

Mean PH of Savitri NILs was significantly reduced under drought stress conditions ranged from 61 to 85 cm in SS, 58 to 75 cm in MS and from 78 to 102 cm in NS conditions. PH was reduced by 17 to 20 cm in drought stress. The mean days to flowering (DTF) in Savitri NILs ranged from 72 to 81 days in SS, 76 to 82 days in MS and from 74 to 81 days in NS conditions. DTF was severely affected under drought for recipient parent Savitri varied from 103 days in NS while 113 and 96 days was recorded in SS and MS conditions respectively. The heritability (H) was high for DTF (0.84,0.85,0.92) and medium to high for PH (0.72,0.80,0.88) and GY (0.74,0.89,0.58) under SS, MS and NS conditions for Savitri NILs (Table 2).

### Promising drought NIL lines with the maximum genetic background coverage of recipient parents

Recurrent parent genome recovery (RPG) were estimated using 111, 107 and 89 polymorphic SSR markers among NILs carrying *qDTYs* in IR64, TDK-*Sub1* and Savitri backgrounds, respectively. The background recovery among the NILs of multiple genetic backgrounds ranged from 85 to 93% in the case of IR64 NILs, 86 to 90% in the case of TDK-*Sub1* NILs and 80 to 89% in the case of Savitri NILs. Most of the IR64 NIL lines showed more than 90% RPG recovery followed by TDK-*Sub1* and least recovered lines belong to Savitri background. Graphical representation of the selected pyramided NILs achieving higher genome recovery of respective backgrounds is depicted in Supplementary Fig. S1 (IR64 NILs with *qDTY*_*2*.*3*_, *qDTY*_*3*.*2*_), Supplementary Fig. S2 (TDK1-*Sub1* NILs with *qDTY*_*6*.*1*_, *qDTY*_*6*.*2*_) and Supplementary Fig. S3 (Savitri NILs with *qDTY*_*3*.*2*_, *qDTY*_*12*.*1*_). The selected promising NILs capturing higher recurrent genome background and yielded well under SS, MS and NS conditions is presented in Table 3. These superior NILs with excellent drought tolerance could be used a drought tolerant line/variety to be released after multi-location evaluation in national and provincial coordinated trials in target countries.

**Table 3:** Selected promising drought NILs identified with maximum recurrent parent genome recovery.

Background	Promising NILs	Parentage	Foreground	RPG recovery %	GY (kg ha^−1^)		
					Severe stress	Moderate stress	Non-stress
IR64	IR 102784:2-42-16-1-1-2	IR 99621-181/IR 99620-158//IRRI 149	*qDTY*_*2*.*3*_ + *qDTY*_*3*.*2*_	93	1426	1860	5430
IR64	IR 102793:1-11-229-3-1-1	IR 99622-312/IR 99620-158//IRRI 149	*qDTY*_*4*.*1*_ + *qDTY*_*12*.*1*_	92	656	1900	5618
IR64	IR 102793:1-11-192-1-1-4	IR 99621-14/IR 99619-361//IRRI 149	*qDTY*_*1*.*2*_ + *qDTY*_*12*.*1*_	91	1386	1110	4748
IR64	IR 102784:2-62-66-1-1-2	IR 99621-181/IR 99620-158//IRRI 149	*qDTY*_*1*.*1*_ + *qDTY*_*1*.*2*_ + *qDTY*_*12*.*1*_	91	611	845	5255
IR64	IR 102796-14-140-1-1-1	IR 99622-306/IR 99620-228//IRRI 149	*qDTY*_*1*.*2*_ + *qDTY*_*12*.*1*_	90	788	2740	4928
IR64	IR 102796-14-124-1-1-3	IR 99622-306/IR 99620-228//IRRI 149	*qDTY*_*2*.*2*_ + *qDTY*_*2*.*3*_	90	996	2235	5220
IR64	IR 102783:2-70-112-3-1-1	IR 99621-14/IR 99619-361//IRRI 149	*qDTY*_*12*. *1*_	90	3375	2210	5143
IR64	IR 102793:1-11-64-1-1-2	IR 99621-181/IR 99620-158//IRRI 149	*qDTY*_*1*.*2*_ + *qDTY*_*12*.*1*_	89	2002	2750	6145
IR64	IR 102793:1-11-66-3-1-1	IR 99622-312/IR 99620-158//IRRI 149	*qDTY*_*1*.*2*_ + *qDTY*_*12*.*1*_	89	2141	1455	4375
TDK1-*Sub1*	IR 102777-5-83-1-2-7	IR 90266-B-542-1/IR07F289	*qDTY*_*6*.*1*_ + *qDTY*_*6*.*2*_	90	316	465	5590
TDK1-*Sub1*	IR 102777-6-86-2-2-11	IR 90266-B-542-1/IR07F289	*qDTY*_*3*.*1*_ + *qDTY*_*6*.*1*_ + *qDTY*_*6*.*2*_	89	479	1555	4966
Savitri	IR 106523-21-28-1-2-B	IR 102773-1061//IR 90252-B-548-2-B/IR 90250-B-475-1-B	*qDTY*_*3*. *2*_	89	517	2565	7098

Note: RPG: Recurrent parent genome recovery.

### QTL–QTL and QTL-background interaction in drought NILs

**Figure 1:**
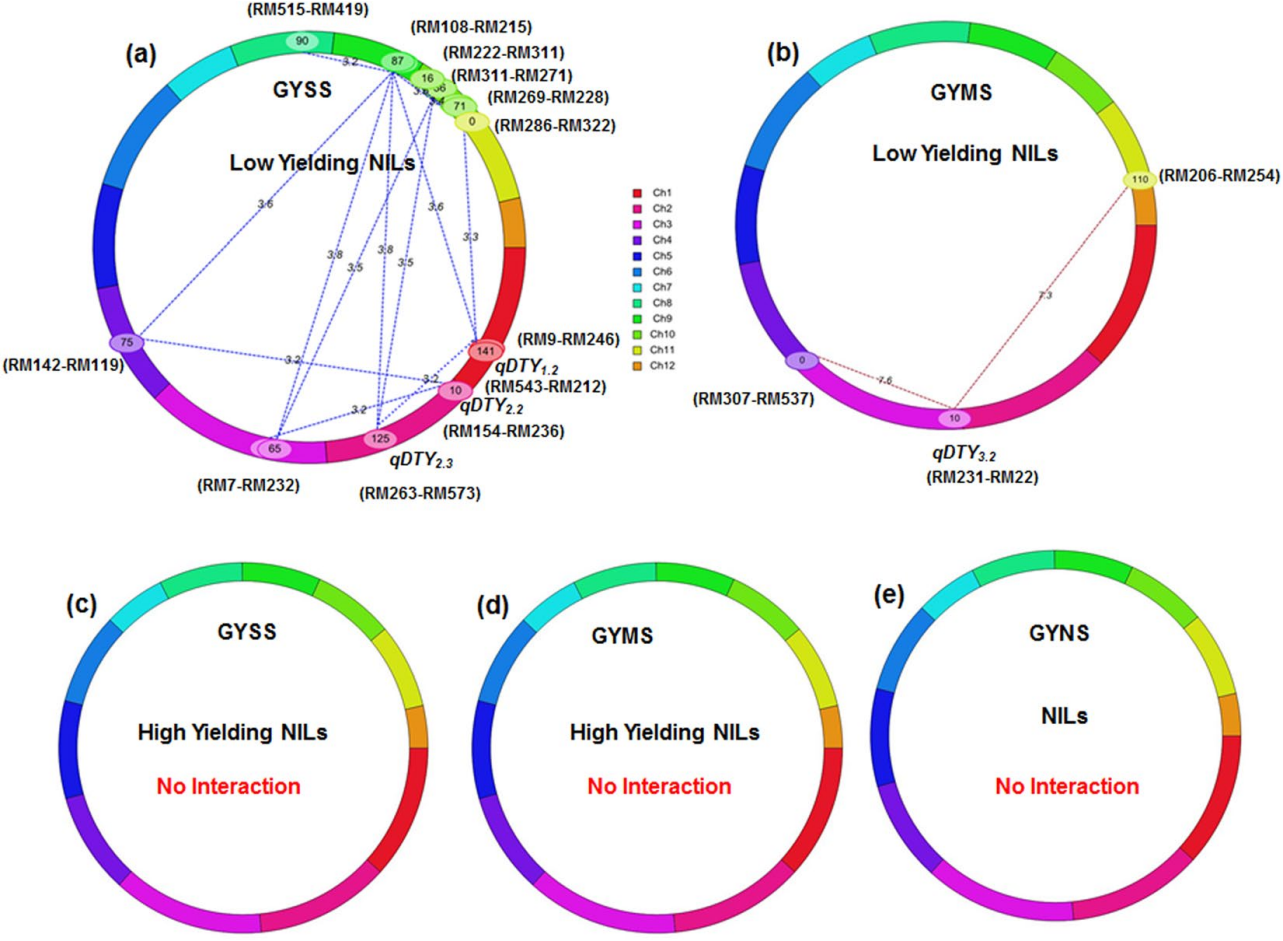
Cyclic illustrations of epistatic QTLs of IR64 NILs for grain yield under various levels of drought stress. The dotted lines indicate marker pairs interacting significantly on same or different chromosomes with their corresponding LOD value due to epistatic effect (**a**) Grain yield under severe stress (GYSS): chromosome 1 (*qDTY*_*1*.*2*_ RM543-RM212) showed epistatic negative interaction with *qDTY*_*2*.*3*_ (RM263-RM573) on chromosome 2 while *qDTY*_*2*.*2*_ (RM154-RM236) and *qDTY*_*2*.*3*_ (RM263-RM573) showed epistatic interaction with background markers loci on chromosomes 3, 4, 9 and 10 (**b**) Grain yield under moderate stress (GYMS): chromosome 3 (*qDTY*_*3.2*_ RM232-RM22, 10 cM) showed epistatic interaction with background loci of chromosomes 4 and 11 (**c**,**d**) GYMS and GYSS of high yielding NILs: no significant interactions was found (**e**) Grain yield under non- stress (GYNS): no interaction.

A total of 16 digenic interactions between marker loci were detected in low yielding NILs of IR64 background under SS and MS situation. Out of 16 digenic interactions, we identified 6 interactions between drought grain yield QTLs (*qDTY*_*2*.*2*_, *qDTY*_*2*.*3*_ and *qDTY*_*3*.*2*_) and background markers loci while 9 interactions were found among background markers loci of different chromosome. A Significant negative QTL-QTL interaction was also found between introgressed *qDTY*_*1*.*2*_ (RM543-RM212) located on chromosome 1 with *qDTY*_*2*.*3*_ (RM263-RM573) identified on chromosome 2. In context of QTL and background marker loci interactions, *qDTY*_*2*.*2*_ (RM154-RM236) interacted negatively with two genetic loci (RM7-RM332) on chromosome 3 and chromosome 4 (RM142-RM119), while *qDTY*_*2*.*3*_ (RM263-RM573) was interacted negatively with genetic loci (RM108-RM215) on chromosome 9 and chromosome 10 (RM271-RM269) under SS situation (Table 4, Fig. 1a). Similarly, *qDTY*_*3*.*2*_ interacted negatively with background markers loci at RM307-RM537 on chromosome 4 and at RM206-RM254 located on chromosome 11 under MS condition (Table 4, Fig. 1b). Out of 9 digenic background markers interaction detected in present study, two negative interactions with marker interval (RM7-RM232; chromosome 3) was found at single loci position at 65 cM with background markers located on chromosomes (9 and10). Similarly, two negative interactions with marker interval (RM246-RM543; chromosome 1) were found at single loci position at 141 cM with background markers located on chromosomes (9 and 11). The PVE% of epistatic QTLs ranged from 3.47 to 25.46% under SS to MS. Surprisingly, none of high yielding NILs expressed any interactions in SS and MS conditions (Fig. 1c,d). Also, no significant interactions were also observed under NS conditions for IR64 NILs including both high yielding as well as low yielding NILs (Fig. 1e). A heat map according to LOD score along the chromosomes reflecting all the significant interactions among low yielding IR64 NILs under SS and MS situations is presented in Supplementary Fig S4.

**Table 4:** Epistatic interaction of major effect loci of grain yield under drought with background loci of IR64, TDK1-*Sub1* and Savitri NILs.

NILs Background	Trait Name	Chromo Some1*	Position 1^†^	Flanking Markers (position1)	Chromo Some 2^‡^	Position 2^⁑^	Flanking Markers (position 2)	LOD epi^§^	PVEepi (%)^¶^	LOD add^§§^	PVE add (%)^¶¶^	Add1^⁑⁑^	Add2^‡‡^	Add1 by Add2^††^
IR64	GYMS	3	10	RM231-RM22 (*qDTY*_*3*.*2*_)	4	0	RM307- RM537	7.67	25.46	3.17	4.89	124.65	458.93	−298.92
IR64	GYMS	3	10	RM231-RM22 (*qDTY*_*3*.*2*_)	11	110	RM206- RM254	7.32	15.64	3.025	3.00	214.96	231.23	−235.02
IR64	GYSS	1	146	RM543-RM212 (*qDTY*_*1*.*2*_)	2	124.7	RM263- RM573 (*qDTY*_*2*.*3*_)	3.20	4.98	2.60	2.57	625.07	−248.3	−972.50
IR64	GYSS	1	141	RM246-RM543	9	86.7	RM108-RM215	3.33	4.75	2.50	2.60	603.78	−269.83	−947.59
IR64	GYSS	1	141	RM246-RM543	11	0	RM286-RM332	3.60	4.66	2.68	2.47	−363.62	579.67	−974.07
IR64	GYSS	2	124.7	RM263- RM573 (*qDTY*_*2*.*3*_)	9	86.7	RM108-RM215	3.82	4.43	2.79	2.77	−396.48	507.01	−990.95
IR64	GYSS	2	9.8	RM154-RM236 (*qDTY*_*2*.*2*_)	3	65	RM7- RM232	3.26	3.92	2.94	2.65	−426.60	481.96	−966.28
IR64	GYSS	2	9.8	RM154-RM236 (*qDTY*_*2*.*2*_)	4	75	RM142-RM119	3.20	4.66	2.68	2.47	−363.62	579.67	−974.07
IR64	GYSS	2	84.8	RM341- RM475	10	21.3	RM222-RM311	3.67	3.54	2.84	2.58	434.01	−424.80	−962.57
IR64	GYSS	2	84.8	RM341-RM475	9	86.7	RM108-RM215	3.75	4.63	2.99	2.60	553.25	−393.57	−992.71
IR64	GYSS	2	124.7	RM263- RM573 (*qDTY*_*2*.*3*_)	10	66.3	RM271-RM269	3.52	4.62	2.74	2.58	−400.88	545.46	−999.2
IR64	GYSS	3	65	RM7-RM232	9	86.7	RM108-RM215	3.81	4.63	2.94	2.59	554.08	−392.93	−991.94
IR64	GYSS	3	65	RM7-RM232	10	36.3	RM311-RM271	3.52	4.43	2.76	2.58	504.44	−394.71	−991.78
IR64	GYSS	4	75	RM142-RM119	9	86.7	RM108-RM215	3.60	4.63	2.92	2.59	553.86	−393.27	−992.10
IR64	GYSS	8	90	RM515-RM419	9	91.7	RM108-RM215	3.27	4.64	2.92	2.58	555.68	−391.84	−992.32
IR64	GYSS	10	16.3	RM222-RM311	10	71.3	RM269-RM228	3.39	3.47	2.68	2.50	−501.44	328.77	−1027.18
TDK1-*Sub1*	GYSS	2	91.9	RM300- RM475	6	10	RM587- RM510 (*qDTY*_*6*.*1*_)	31.06	14.09	13.302	4.09	−73.18	72.46	−72.46
TDK1-*Sub1*	GYSS	3	150	RM411- RM319	6	10	RM587- RM510 (*qDTY*_*6*.*1*_)	30.38	12.40	11.162	5.80	−22.97	50.06	−26.05
TDK1-*Sub1*	GYSS	5	36.7	RM592- RM437	6	10	RM587- RM510 (*qDTY*_*6*.*1*_)	30.40	17.90	13.89	3.93	−122.05	113.11	−113.11
TDK1-*Sub1*	GYSS	6	10	RM587- RM510 (*qDTY*_*6*.*1*_)	6	115	RM275- RM30	28.0	14.88	10.87	4.98	45.55	−42.51	−44.08
TDK1-*Sub1*	GYSS	6	10	RM587- RM510 (*qDTY*_*6*.*1*_)	11	100	RM473-RM206	30.14	19.95	14.30	4.76	40.54	−35.12	−35.17
Savitri	GYMS	4	8.5	RM537-RM335	6	65	RM136-RM275	3.43	10.56	2.41	3.90	2.62	−211.62	−382.62
Savitri	GYMS	10	15	RM244-RM239	6	65	RM136-RM275	3.43	12.76	2.58	4.22	−211.62	2.62	−382.62
Savitri	GYSS	2	49.8	RM71- RM290	3	182	RM55- RM570	4.31	10.78	2.80	5.01	10.66	−58.66	−238.33
Savitri	GYSS	3	67	RM7- RM251	3	187	RM55- RM570	4.10	12.76	2.61	7.94	−58.66	10.66	238.33
Savitri	GYSS	3	167	RM319- RM55	7	73.2	RM10- RM47	6.33	14.31	2.44	2.12	−69.38	144.08	−94.60
Savitri	GYSS	3	182	RM55- RM570	11	0	RM4B- RM332	4.21	11.78	2.88	4.01	−58.66	10.66	−238.33

Note: GYMS: Grain yield under moderate stress; GYSS: Grain yield under severe stress; ^*^Chromosome ID at the first scanning position; ^†^Scanning position in cM of the first flanking marker pair; ^‡^Chromosome ID at the second scanning position; ^⁑^Scanning position in cM of the second flanking marker pair; ^§^LOD score caused by epistatic effects; ^¶^PVE(%): Phenotypic variation explained by epistatic effects; ^§§^LOD score caused by additive effects; ^¶¶^PVE(%): Phenotypic variation by the additive effects; ^⁑⁑^Estimated additive effect of position 1; ^‡‡^Estimated additive effect of position 2; ^††^Additive by additive epistatic effect at the two scanning positions.

**Figure 2:**
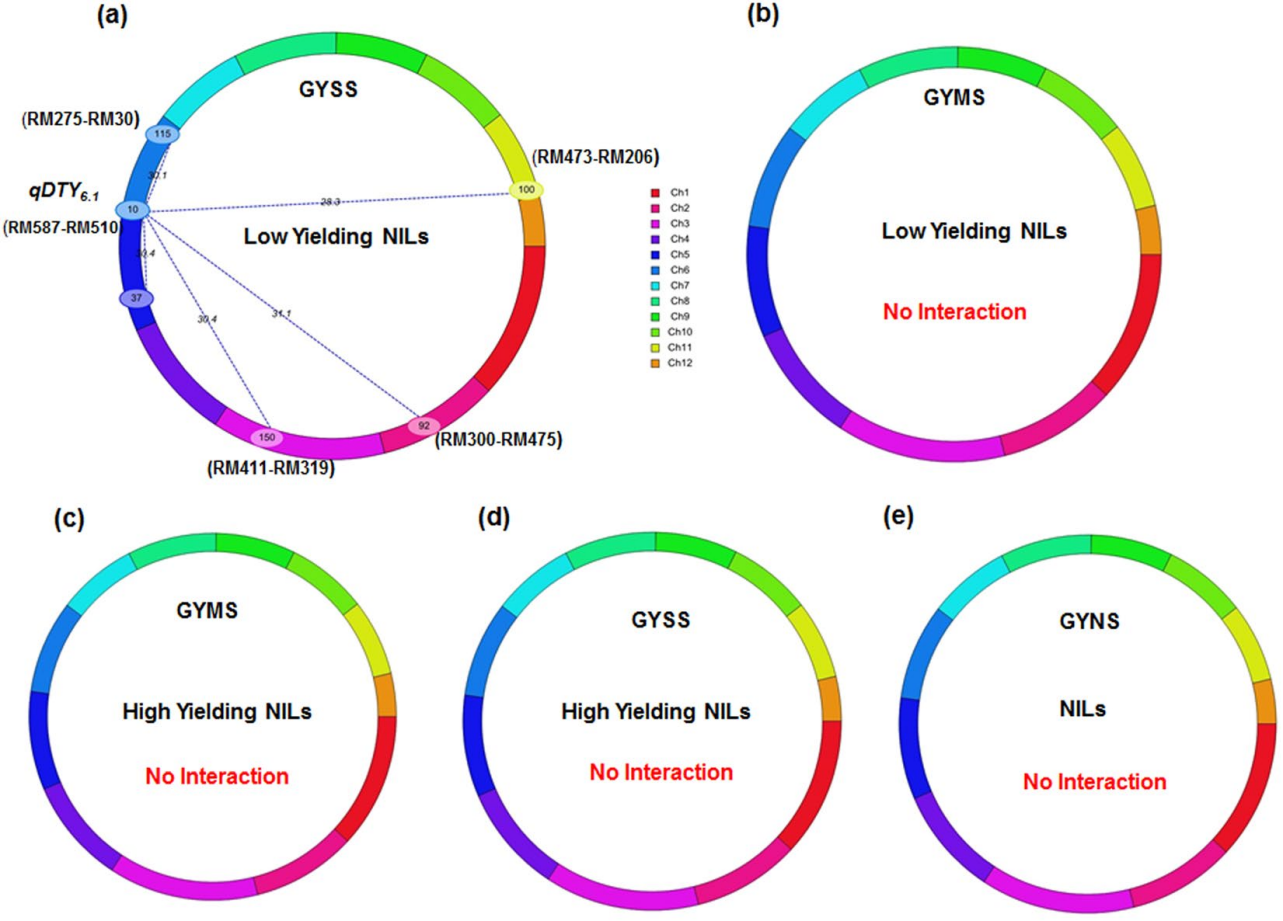
Cyclic illustrations of epistatic QTLs of TDK1-*Sub1* NILs for grain yield under various levels of drought stress (**a**) Grain yield under severe stress(GYSS) Chromosome 6 (*qDTY*_*6*.*1*_ RM587-RM510,10 cM) showed epistatic interaction with background loci of chromosomes 2, 3, 5 and 11 (**b**) Low yielding GYMS: no interaction, (**c**,**d**) GYMS and GYSS of high yielding NILs: no significant interactions was found (**e**) GYNS: no interaction.

**Figure 3:**
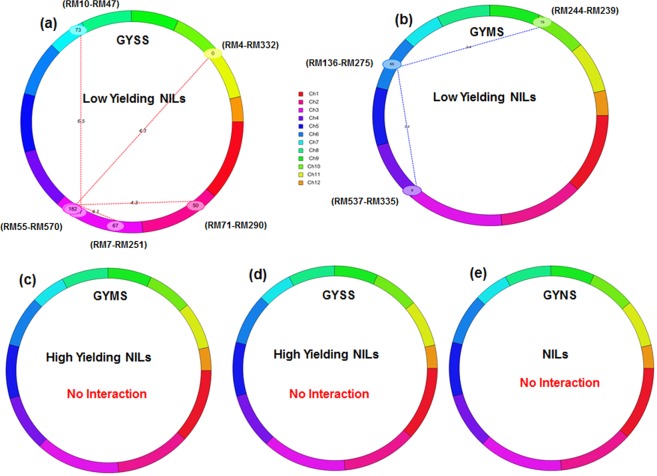
Cyclic illustrations of epistatic QTLs of Savitri NILs for grain yield under various levels of drought stress (**a**) GYSS: chromosome 2 (RM71-RM290,50 cM) and chromosome 3 (RM7-RM251 67 cM, RM319-RM55 167 cM and RM55-RM570,182 cM) have shown epistatic interaction with background loci of chromosomes 3,7 and 11 (**b**) GYMS: chromosome 4(RM537-RM335) and chromosome 10(RM244-RM239) was interacted negatively with a common genetic loci (RM136-RM275) on chromosome 6 (**c**,**d**) GYMS and GYSS of high yielding NILs: no significant interactions was found (**e**) GYNS: no interaction.

Low yielding NILs of TDK1-*Sub1* background had shown 5 significant epistatic interactions and all these interactions were detected between introgressed QTL *qDTY*_*6*.*1*_ (RM587-510) and background markers loci located across chromosomes 2, 3, 5, 6 and 11. Two negative interactions were found between QTL *qDTY*_*6*.*1*_ (RM587-510) and marker pairs RM115-RM275 and RM473-RM206 located on chromosomes 6 and 11 respectively while 3 interactions were found between background markers loci (RM300-RM475 on chromosome 2; RM411-RM319 on chromosome 3; RM592-RM437 on chromosome 5) with a common loci (RM587-510) associated with *qDTY*_*6*.*1*_ (Table 4, Fig. 2a). The PVE% of interacting QTLs ranged from 12.04 to 19.95%. Low yielding NILs under MS (Fig. 2b) and high yielding NILs under SS (Fig. 2c) and MS (Fig. 2d) have not shown any significant interactions. Also, no significant interactions were observed under well-watered (NS) conditions for TDK1-*Sub1* NILs including both high yielding and low yielding NILs (Fig. 2e). A heat map according to LOD score along the chromosomes reflecting the significant digenic interactions found among low yielding TDK1-*Sub1* NILs under SS situations is presented in Supplementary Fig S5. In total, 6 epistatic interactions were identified among background markers loci under SS and MS in low yielding lines of Savitri background (Table 4, Fig. 3a,b). The interaction between introgressed QTLs (*qDTY*_*3*.*2*_, *qDTY*_*12*.*1*_) and background markers loci was not identified in this background. Despite, away from position of introgressed QTL *(qDTY*_*3*.*2*_; 10 cM) flanking markers (RM7-RM251, 67 cM) interacted negatively with background marker pair RM55-570 on chromosome 3 under SS situation. Under MS, on chromosome 4 (RM537- RM335) and chromosome 10 (RM244- RM239) were interacted with a common genetic locus (RM136- RM275) on chromosome 6 with explaining PVE upto 12.76%.No interactions were found in high yielding NILs under SS and MS conditions (Fig. 3c,d). Also, no significant interactions were found under NS condition for Savitri NILs including both high yielding as well as low yielding NILs (Fig. 3e). A heat map according to LOD score along the chromosomes reflecting the significant digenic interactions found among low yielding Savitri NILs under SS and MS situations is presented in Supplementary Fig S6.

## Discussion

Epistasis is a genetic phenomenon of interaction which may enhance or reduce the expression (depending on degree and direction) of interacting loci underlying QTLs associated with the complex trait^44,45^. QTL mapping and interaction studies in recent years in different crops suggested the possible presence of epistasis such as (1) interactions between introgressed QTLs (2) interactions between introgressed QTLs and ‘background’ (modifying) loci and (3) interactions between complementary background loci^46^ affecting overall expressions of trait. To further understand this interaction (QTL-QTL and QTL to background), we have selected range of rice drought NILs developed in different genetic backgrounds.

Several large and consistent effect QTLs for grain yield under drought (*qDTY*_*1*.*1*_, *qDTY*_*2*.*2*_, *qDTY*_*3*.*1*_, *qDTY*_*12*.*1*_) were identified and validated in various rice genetic backgrounds using the QTL associated markers^4,6,7,9^. Exploiting the available molecular markers for introgression of consistent drought grain yield QTLs (*qDTYs*) through MAS is most desirable step for generation of drought-tolerant lines/varieties. In this study, contrasting performance of a pair of NILs possessed similar *qDTY* and *qDTY* combinations were analyzed to identify the possible epistatic interactions of introgressed *qDTYs* QTLs and background marker loci under different level of stress and non -stress conditions.

Genetic backgrounds IR64, TDK1-*Sub1* and Savitri, used in this study to develop NILs which produces higher grain yield under drought stress condition, widely cultivated and accepted by farmers in India, Lao PDR and Nepal, respectively. Foreground selection using the flanking markers associated with *qDTYs* QTLs ensured successful incorporation of the target QTLs in NILs of respective backgrounds (Supplementary Table S1). Marker-assisted background selection using uniformly distributed polymorphic markers have been extensively used to track recovery of background genome^19,47,48^. Rice drought breeding has witnessed some of the successful MAS introgression of *qDTYs* QTLs in elite backgrounds^7,8,12,13^ using SSR and SNP markers for background selection. The Broad-sense heritability estimates in Tables 1, 2 were recorded moderate to high for grain yield in all the experiments varied from 0.58 (58%) to 0.89 (89%). Many of our previous studies have also reported moderate to high heritability for grain yield under drought stress for QTL identification^4,8^. In present study, 600 genome-wide SSR markers were used for estimating the similarity between backgrounds and their derived NILs. Promising NILs have captured 89–93% recovery of respective backgrounds (Table 3) while 44 NILs used in this study possessed 85–93% genome recovery. Phenotypic selection combined with marker-assisted background selection approach after *qDTY* introgression was followed, that could be one of the reasons not to achieve the expected recovery in some of the NILs. Similar concern was discussed earlier by Dixit *et al*.^49^ in his study of marker-assisted breeding to combine submergence and drought tolerance in rice. However, in future drought NILs advancement, donor segment can be further minimized by using the recombinant selection markers and generation of whole genome profiling data through SNP-chips^19^ of respective *DTY* QTLs. A similar approach was also explained by Jena *et al*.^25^ in context to rice NILs developed for brown planthopper (BPH) resistance having low recovery (82–89.5%) of recurrent parent genome IR24 even after three back crosses.

Role of digenic interactions between *DTY* QTLs affecting the performance of one QTL over the other drought QTL has been well documented in various QTL mapping and marker-assisted drought breeding programme^12,42,43^. Positive interaction of *qDTY*_*12*.*1*_ with *qDTY*_*2*.*3*_ and *qDTY*_*3*.*2*_ have been reported by Dixit *et al*.^42^ which increases grain yield under drought significantly while Shamsudin *et al*.^12^ identified positive digenic interactions of *qDTY*_*2*.*2*_ and *qDTY*_*3*.*1*_ with *qDTY*_*12*.*1*_ enhancing overall expression of drought-related traits. Interaction of *qDTY*_*3*.*2*_ with *qDTY*_*1*.*1*_ and *qDTY*_*12*.*1*_ reduces the flowering duration and subsequently increases GY under stress^43^. A positive epistatic interaction of *qDTY*_*4*.*1*_ and *qDTY*_*9*.*1*_ loci with *qDTY*_*7*.*1*_ have been reported in the pyramided line of Samba Mahsuri enhancing the grain yield under drought^28^. Apart from these known *qDTYs* QTL x QTL interactions, there are many unknown genomic interactions exist with background loci called as background noise which should be identified for effective deployment of drought QTLs in MAS breeding program.

Interaction between the target QTL/gene with the genetic background has been discussed earlier for various complex traits in rice^18,50,51^. However, there have been limited attempts to identify particular background markers loci interacting with introgressed QTLs and influencing the overall expression of target trait. There is an urgent need to identify such epistatic gene interactions which complicate the genotype-phenotype relationship of complex traits such as drought. In present study, we have detected negative interactions between drought QTLs (*qDTY*_*2*.*2*_, *qDTY*_*2*.*3*_ and *qDTY*_*3*.*2*_) with IR64 background markers loci located on chromosomes (2, 3, 4, 10 and 11), among background markers loci located on different chromosomes and between drought grain yield QTL (*qDTY*_*1*.*2*_) with *qDTY*_*2*.*3*_, which might be one of the reason of poor performance of low yielding NILs carrying DTY QTLs (Table 1). The influence of epistatic drought QTL interactions explained PVE% ranging from 3.47% to 25.46% which could be very crucial in determining the grain yield under drought. Five digenic interactions were found in low yielding NILs of TDK1-*Sub1* background, where *qDTY*_*6*.*1*_ (RM587-RM510) had shown negative interaction with background marker located on five different chromosomes. Savitri background NILs had shown 6 epistatic interactions between background markers loci in low yielding lines. Many of such epistatic interactions are background specific. However, in our study, the high effect of many of the *qDTY* QTLs interaction with the genetic background marker loci indicate that these are biological interactions and not just statistical. The QTLs *qDTY*_*2*.*2*_, *qDTY*_*2*.*3*_, *qDTY*_*3*.*2*_ and *qDTY*_*6*.*1*_ have shown interactions with the background markers on the same chromosome in three other genetic backgrounds (data unpublished).Some earlier studies^32,52^ highlighted the importance of epistatic QTL effect over the additive QTLs are influencing the expression of introgressed trait in crops like Arabidopsis and rice. Thomson *et al*.^53^ reported a loss of *Saltol* QTL effect in developed NILs and suggested an interaction between *Saltol* and other background loci. Similarly, Babu *et al*.^54^ observed the difference in level of seedling stage salt tolerance among *Saltol* introgressed advanced lines and assumed the existence of a possible interaction between *Saltol* QTL and genetic background but these reports did not report on the particular background markers loci interacting negatively with *Saltol* QTL, resulting in variable expression of *Saltol* introgressed lines. Revealing such epistatic interactions in present study, between introgressed QTL and background markers loci sheds more light in understanding the differences in phenotypic expression of MAS introgressed/pyramided lines for quantitative traits that can be useful in future MAS programs.

## Conclusions

Performance of NILs carrying same major effect drought grain yield QTLs/QTLs combinations are strongly affected by interactions with the genetic background loci. This is the first report on in-depth analysis of loss of introgressed QTL effects in SS, MS due to negative interactions between drought QTLs and background markers loci, drought QTL-QTL interaction and among interacting background markers loci on various chromosomes in SS, MS in low yielding NILs but not under NS. For both low as well as high yielding NILs, no interaction was detected under NS. Absence of such negative interactions in high yielding NILs under MS, SS indicate future MAS program to carefully select against such interactions to increase grain yield under drought. Promising NILs in background of popular varieties (IR64, TDK1-*Sub1* and Savitri) with absence of negative interactions and performing well in drought stress and non-stress conditions might be a very useful lines for use in future MAS/variety to be released for drought-prone ecosystems.

## Methods

### Plant materials

A total of 44 drought NILs derived from three backgrounds (33 NILs of IR64, 5 NILs of TDK1-*Sub1* and 6 NILs of Savitri) were used in this study (Supplementary Table S2). We initiated the process of NILs development with a total of 130 lines (70 lines from IR64, 32 lines from TDK1-*Sub1* and 28 lines from Savitri) developed through marker-assisted breeding for the three genetic backgrounds for the present study. However, before including all the lines we attempted foreground selection with QTLs linked markers as well as background marker selection of all the lines with 600 genome wide background SSR markers and those lines which follows the minimum criteria of RPG recovery (>80%) were taken for the selection of NILs for further evaluation. As a result, finally we have selected a total of 44 drought NILs derived from three backgrounds (33 NILs of IR64, 5 NILs of TDK1-*Sub1* and 6 NILs of Savitri) were used in this study. A total of eight sets of IR64 NILs (20 high yielding and 13 low yielding) possessing different QTL and QTL combinations was developed through crossing of IR64 with different drought tolerant donors contributing different grain yield QTLs. Four different drought tolerant donors were utilized for development of the selected NILs, for instance IR77298-14-1-2-17 (*qDTY*_*2*.*2*_ and *qDTY*_*4*.*1*_), IR 86918-B-315 (*qDTY*_*1*.*1*_ and *qDTY*_*1*.*2*_), Vandana (*qDTY*_*2*.*3*_ and *qDTY*_*3*.*2*_) and Way Rarem (*qDTY*_*12*.*1*_) were crossed with IR64 and through marker assisted backcross breeding the above mentioned NILs were developed^7^. The pyramided version of NILs was developed through intercrossing of NILs of different gene combinations^7^. Two sets of TDK1-*Sub1* NILs (2 high yielding and 3 low yielding NILs) possessing different QTL combinations were utilized in the present study. The pyramided version of NILs were developed from the cross between IR55419-04/2*TDK1. After confirming the presence of three QTLs (*qDTY*_*3*.*1*_, *qDTY*_*6*.*1*_ and *qDTY*_*6*.*2*_) in pyramided line, it was utilized for backcrossing with submergence tolerant parent TDK1-*Sub1* to develop NILs in TDK1-*Sub1* background^8,49^. Similarly, two sets of Savitri NILs (3 high yielding and 3 low yielding NILs) were developed from intercrossing of two BC_1_ mapping population (IR 77298-5-6-18/2*Savitri IR 74371-46-1-1/2*Savitri) to develop lines carrying *qDTY*_*3*.*2*_ and *qDTY*_*12*.*1*_ QTLs in Savitri background^10,11,13^. All of the NILs used in the present study in all the three background were re-confirmed with earlier reported markers for the presence of QTL and QTL combinations.

### Evaluation of NILs under reproductive-stage drought stress and non-stress conditions

To study the grain yield variability of NILs pyramided with same QTL and QTL combinations, a total of 33 NILs derived from IR64, 5 NILs from TDK1-*Sub1* and 6 NILs from Savitri backgrounds were evaluated during the dry seasons (DS) of 2015 and 2017 in a transplanted lowland ecosystem under reproductive-stage drought stress (RS) and non-stress (NS) conditions at the Ziegler Experiment Station of IRRI, Los Baños, Laguna, Philippines (14 °30′N longitude 121 °15′E latitude). Seedlings were raised in a wet-bed nursery for 21 days before transplanting into the main experimental field. The field lay out was generated using the statistical software Plant Breeding Tools (PBTools v1.4) developed at IRRI. The experimental design was alpha lattice in 2 replications in two-row plots of 5 m length with row to row and plant to plant spacing of 20 × 20 cm. Continuous supply of irrigation at 5 cm standing water was provided till the crop maturity in non-stress experiment. Fertilizer nitrogen, phosphorus, and potassium (NPK) were applied at recommended dose of 120:30:30 kg ha^−1^. In RS trials, two doses of nitrogen fertilizer (basal and first split) were applied before initiating stress and a third dose was applied in adjustment with life-saving irrigation.

Reproductive stage drought stress experiment was conducted as described previously^4,6^. In brief for RS experiment normal irrigation was maintained in the field up to 30 days after transplanting, after which the water was drained out to initiate stress and continued till the harvesting. Water table depth was measured using PVC pipe with a plugged hole at the bottom and inserted in soil to 1 m depth and 15 cm above the soil surface in experimental stress field at regular interval. Life-saving irrigation was provided at level of severe stress when all the susceptible checks showed severe leaf rolling with minimum probability to recover upon watering and water table remained below 1 meter. Life-saving irrigation provided through flash flooding was drained out after 24 hrs to initiate further cycle of stress.

The stress imposed was classified from severe to moderate based on the relative yield reduction to non-stress (NS) experiments explained earlier by^42,55^. Experiments with yield reduction of <70% were characterized as severe stress (SS) and experiments with yield reduction of 31–70% were characterized as moderate stress (MS) as earlier reported by Kumar *et al*.^55^.

### Observations recorded

Data on days to 50% flowering (DTF), plant height (PH), and grain yield (GY) were recorded in RS and NS experiments. DTF was observed as the number of days from seeding to the 50% the plants in a plot flower. The PH of three random plants from each NILs was measured before maturity from the soil surface to the tip of the main tiller and then averaged for mean analysis. Grains from individual plot were harvested at physiological maturity, oven-dried to a moisture content of 14% before weighing^6,9^. The grain yield in grams was further converted in kg ha^−1^ before entry means analysis in PBTools.

### NILs genotyping

The presence of introgressed drought QTLs and QTLs combinations in IR64 (*qDTY*_*1*.*1*_, *qDTY*_*1*.*2*_, *qDTY*_*2*.*2*_, *qDTY*_*2*.*3*_, *qDTY*_*3*.*2*_, *qDTY*_*4*.*1*_ and *qDTY*_*12*.*1*_) TDK1-*Sub1* (*qDTY*_*3*.*1*_, *qDTY*_*6*.*1*_, and *qDTY*_*6*.*2*_) and Savitri backgrounds (*qDTY*_*3*.*2*_ and *qDTY*_*12*.*1*_) were confirmed in respective NILs using the earlier reported peak and flanking markers of respective grain yield drought QTLs^4,6–9^. Fresh and young leaves from 25 days old transplanted seedlings were collected in plot-wise bulk from each NIL- and their respective parents. Genomic DNA was extracted from collected leaves using a modified CTAB protocol developed by Murray and Thompson^56^, dissolved in 200 μl of TE (Tris-EDTA) buffer and stored at −20 °C. PCR amplification with the molecular markers was performed on a thermal cycler (G-Storm GS1, UK) at Genotyping service laboratory (GSL), IRRI, Philippines. Each PCR reaction mixture of 15 μl consisted of 10 ng of rice genomic DNA, 1 × PCR buffer, 100 μM dNTPs, 100 μM oligonucleotide primers and 1 unit of Taq polymerase. PCR products were resolved casting high-resolution 8% (v/v) polyacrylamide gel electrophoresis (PAGE) (CBS scientific, model MGV-202–33) and resolved in a 1x TBE buffer at 90 volts for 1–2 h, depending on the PCR product sizes. The separated DNA fragments after electrophoresis were stained with SYBER Safe™ and visualized under UV trans-illuminator (AlphaImager™ System).

To study the genomic reconstitution of NILs with the similar QTL or QTLs combinations but showing variation in grain yield under drought, background genotyping was carried out on the selected NILs and parents. A total of 600 SSR markers equally distributed on all 12 chromosomes were selected from the Gramene database (http://www.gramene.org/) for identification of polymorphic markers between parents, which were further utilized for background selection in order to estimate recurrent parent genome (RPG) recovery.

### Statistical analysis, recurrent parent genome recovery and epistatic interaction analysis

The phenotypic data collected from drought stress and non-stress experiments were statistically analyzed for the computation of trial means and standard error of difference (SED) using PBTools v1.4. Least significant difference (LSD) “at the 5% and 1% significant levels” were used to compare the means of the test entries and to infer the significant differences of the traits studied between parents and each NILs. Linear mixed model for analysis of variance was calculated with following equation:

$$Yijk={\mu }+Gi+Rj+BK(Rj)+eijk$$

where, Yijk is measurement recorded in plot, µ is overall mean, Gi is effect of i^th^ genotype, Rj is the effect of the j^th^ replicate, BK (Rj) is the block effect of j^th^ replicate and eijk is the error. The genotypes were considered as fixed and the replicates and block effects were random for estimating the entry means. The recurrent parent genome (RPG) recovery percentage of drought NILs of various background were estimated with the following formula^57^

$${\rm{Recurrent}}\,{\rm{parent}}\,{\rm{genome}}\,({\rm{RPG}})\,{\rm{recoery}}\, \% =\frac{2({\rm{B}})+({\rm{H}})}{2N}\times 100$$

Where, B=SSR marker loci homozygous for genetic background; H = no. of marker loci still in heterozygous; N = total no. of polymorphic SSR markers used for background estimation.

Graphical representation of background genome was constructed using software Graphical Genotype (*GGT v 2*.*0*)^58^ (https://www.plantbreeding.wur.nl/).

Epistatic (digenic) interactions between markers loci of all the NILs including high and low yielding NILs background wise were analyzed considering all the conditions (SS, MS and NS) in model using *QTL IciMapping*^59^ *ver*. *4*.*0*.*1*. In epistasis mapping method, a two-stage stepwise regression strategy was adopted to identify the most significant markers and marker-pair followed by two-dimensional scanning to identify significant digenic epistasis using adjusted phenotypic values based on best fitted multiple regression model^60^. In the first stage, the significant markers and marker pairs explaining epistatic and additive variations were selected in model and then in next stage the stepwise regression with stricter probability levels were applied to the residuals from the first stage to select significant marker pairs and estimate their effects in model^50.^ The stricter probability level was applied in the second stage to avoid over-fitting due to the large number of regression variables. Extensive simulations have shown epistatic QTLs and QTL epistatic networks can be identified efficiently by ICIM mapping even in case two interacting QTL have some additive effects^60^. The threshold LOD value was determined by a permutation test involving 1000 runs at a significance level of P = 0.01 to detect significant digenic interactions between marker loci. In mapping parameters, window size and walk speed used for the genome scan was 10 cM and 1 cM respectively.

## Supplementary information

Supplementary material

## Data Availability

The data sets supporting the results of this article are included within the article.
