# Supplementary material for: Epistatic interactions of major effect drought QTLs with genetic background loci determine grain yield of rice under drought stress
Source: Sci Rep. 2019 Feb 22;9:2616. doi: 10.1038/s41598-019-39084-7 (PMC6385343; doi:10.1038/s41598-019-39084-7)
Supplement: Supplementary file 1 — Supplementary material [file 41598_2019_39084_MOESM1_ESM.pdf]

Epistatic interactions of major effect drought QTLs with genetic background loci determine grain yield of rice under drought stress

Shailesh Yadav[1] , Nitika Sandhu[1], Ratna Rani Majumder[1], Shalabh Dixit[1], Santosh Kumar[2], S.P. Singh[3], N.P. Mandal[4], S. P. Das[5], Ram Baran Yadaw[6], Vikas Kumar Singh[7], Pallavi Sinha[8] , Rajeev K. Varshney[8] and Arvind Kumar[1*]

[1] Rice Breeding Platform, International Rice Research Institute, DAPO Box 7777, Metro Manila, Philippines
[2] ICAR Research Complex for Eastern Region, Patna, Bihar
[3] Bihar Agricultural University, Sabour, Bihar
[4] Central Rainfed Upland Rice Research station, National Rice Research Institute, Hazaribagh, Jharkhand
[5] ICAR Research Complex for NEH Region, Tripura Centre, Lembucherra, Tripura
[6] Regional Agriculture Research Station, NARC, Bara, Nepal
[7] International Rice Research Institute, South Asia Hub, ICRISAT, Patancheru, Hyderabad, India
[8] International Crops Research Institute for the Semi-arid Tropics, Patancheru, Hyderabad, India

[*]**Corresponding author**: a.kumar@irri.org

Supplementary Table S1. Linked markers of introgresed QTLs used in foreground selection of drought NILs

NILs	QTLs with marker interval	Background
IR 102793:1-11-66-3-1-1	$qDTY_{1.2}$ (RM212, RM3825, RM315)	IR64
	$qDTY_{12.1}$ (RM28048, RM28099, INDEL8, RM28130)	
IR 102796-14-132-1-1-3	$qDTY_{1.2}$ (RM212, RM3825, RM315)	IR64
	$qDTY_{12.1}$ (RM28048, RM28099, INDEL8, RM28130)	
IR 102796-14-140-1-1-1	$qDTY_{1.2}$ (RM212, RM3825, RM315)	IR64
	$qDTY_{12.1}$ (RM28048, RM28099, INDEL8, RM28130)	
IR 102793:1-11-192-1-1-4	$qDTY_{1.2}$ (RM212, RM3825, RM315)	IR64
	$qDTY_{12.1}$ (RM28048, RM28099, INDEL8, RM28130)	
IR 102783:2-70-112-2-1-4	$qDTY_{12.1}$ (RM28048, RM28099, INDEL8, RM28130)	IR64
IR 102783:2-70-112-3-1-1	$qDTY_{12.1}$ (RM28048, RM28099, INDEL8, RM28130)	IR64
IR 102784:2-42-88-2-1-2	$qDTY_{12.1}$ (RM28048, RM28099, INDEL8, RM28130)	IR64
IR 102784:2-42-99-2-1-3	$qDTY_{12.1}$ (RM28048, RM28099, INDEL8, RM28130)	IR64
IR 102784:2-90-385-1-1-3	$qDTY_{12.1}$ (RM28048, RM28099, INDEL8, RM28130)	IR64
IR 102783:2-70-135-4-1-1	$qDTY_{12.1}$ (RM28048, RM28099, INDEL8, RM28130)	IR64
IR 102783:2-70-139-2-1-2	$qDTY_{12.1}$ (RM28048, RM28099, INDEL8, RM28130)	IR64
IR 102784:2-62-434-1-1-1	$qDTY_{12.1}$ (RM28048, RM28099, INDEL8, RM28130)	IR64
IR 102784:2-90-385-3-1-1	$qDTY_{12.1}$ (RM28048, RM28099, INDEL8, RM28130)	IR64
IR 102784:2-62-66-1-1-2	$qDTY_{1.1}$ (RM11943, RM12023,RM12233)	IR64
	$qDTY_{1.2}$ (RM212, RM3825, RM315)	
	$qDTY_{12.1}$ (RM28048, RM28099, INDEL8, RM28130)	
IR 102784:2-118-22-1-1-2	$qDTY_{1.1}$ (RM11943, RM12023,RM12233)	IR64
	$qDTY_{1.2}$ (RM212, RM3825, RM315)	
	$qDTY_{12.1}$ (RM28048, RM28099, INDEL8, RM28130)	
IR 102793:1-11-64-1-1-2	$qDTY_{1.2}$ (RM212, RM3825, RM315)	IR64
	$qDTY_{12.1}$ (RM28048, RM28099, INDEL8, RM28130)	
IR 102793:1-11-66-3-1-1	$qDTY_{1.2}$ (RM212, RM3825, RM315)	IR64
	$qDTY_{12.1}$ (RM28048, RM28099, INDEL8, RM28130)	
IR 102784:2-42-17-2-1-1	$qDTY_{2.2}$ (RM154, RM236, RM279, RM555, OSR17, RM492)	IR64
IR 102784:2-42-17-3-1-1	$qDTY_{2.2}$ ((RM154, RM236, RM279, RM555, OSR17, RM492)	IR64
IR 102784:2-42-3-1-1-2	$qDTY_{2.2}$ (RM154, RM236, RM279, RM555, OSR17, RM492)	IR64
	$qDTY_{2.3}$ (RM3212, RM573, RM1367)	
IR 102796-14-124-1-1-3	$qDTY_{2.2}$ (RM154, RM236, RM279, RM555, OSR17, RM492)	IR64
	$qDTY_{2.3}$ (RM3212, RM573, RM1367)	
IR 102784:2-42-136-1-1-3	$qDTY_{2.2}$ (RM154, RM236, RM279, RM555, OSR17, RM492)	IR64
	$qDTY_{2.3}$ (RM3212, RM573, RM1367)	
	$qDTY_{3.2}$ (RM523, RM22, RM545)	
IR 102784:2-42-127-3-1-1	$qDTY_{2.3}$ (RM3212, RM573, RM1367)	IR64
IR 102784:2-42-136-2-1-1	$qDTY_{2.3}$ (RM3212, RM573, RM1367)	IR64
IR 102784:2-42-138-1-1-1	$qDTY_{2.3}$ (RM3212, RM573, RM1367)	IR64
IR 102793:1-11-69-1-1-3	$qDTY_{2.3}$ (RM3212, RM573, RM1367)	IR64
IR 102784:2-118-15-1-1-2	$qDTY_{4.1}$ (RM518, RM335, RM16368)	IR64
IR 102793:1-11-189-2-1-2	$qDTY_{4.1}$ (RM518, RM335, RM16368)	IR64
IR 102784:2-118-549-2-1-2	$qDTY_{4.1}$ (RM518, RM335, RM16368)	IR64
	$qDTY_{12.1}$ (RM28048, RM28099, INDEL8, RM28130)	
IR 102793:1-11-229-3-1-1	$qDTY_{4.1}$ (RM518, RM335, RM16368)	IR64
	$qDTY_{12.1}$ (RM28048, RM28099, INDEL8, RM28130)	
IR 102784:2-62-481-2-1-1	$qDTY_{2.3}$ (RM3212, RM573, RM1367)	IR64
	$qDTY_{3.2}$ (RM523, RM22, RM545)	
IR 102784:2-42-16-1-1-2	$qDTY_{2.3}$ (RM3212, RM573, RM1367)	IR64
	$qDTY_{3.2}$ (RM523, RM22, RM545)	
IR 102784:2-89-284-1-1-3	$qDTY_{4.1}$(RM518, RM335, RM16368)	IR64
	$DTY_{12.1}$ (RM28048, RM28099, INDEL8, RM28130)	

IR 102784:2-89-284-3-1-1	$qDTY_{4.1}$ (RM518, RM335, RM16368)<br>$DTY_{12.1}$(RM28048, RM28099, INDEL8, RM28130)	IR64
IR 102774-26-8-3-2-5	$qDTY_{6.1}$ (RM204, RM217, RM508, RM586, RM587)<br>$qDTY_{6.2}$ (RM3, RM541)	TDK1-*Sub1*
IR 102777-5-83-1-2-7	$qDTY_{6.1}$ (RM204, RM217, RM508, RM586, RM587)<br>$qDTY_{6.2}$ (RM3, RM541)	TDK1-*Sub1*
IR 102777-6-86-2-2-11	$qDTY_{3.1}$ (RM55, RM168, RM186, RM293, RM468)<br>$qDTY_{6.1}$ (RM204, RM217, RM508, RM586, RM587)<br>$DTY_{6.2}$ (RM3, RM541)	TDK1-*Sub1*
IR 102774-15-32-3-1-2	$qDTY_{3.1}$ (RM55, RM168, RM186, RM293, RM468)<br>$qDTY_{6.1}$ (RM204, RM217, RM508, RM586, RM587)<br>$qDTY_{6.2}$ (RM3, RM541)	TDK1-*Sub1*
IR 102777-6-86-2-2-7	$qDTY_{6.1}$ (RM204, RM217, RM508, RM586, RM587)<br>$qDTY_{6.2}$ (RM3, RM541)	TDK1-*Sub1*
IR 106523-21-28-1-2-B	$qDTY_{3.2}$ (RM231, RM517)	Savitri
IR 106531-4-22-3-3-B	$qDTY_{3.2}$ (RM231, RM517)	Savitri
IR 106522-41-8-3-B	$qDTY_{3.2}$ (RM231, RM517)<br>$qDTY_{12.1}$ (RM28048, RM511, RM28199, RM28166)	Savitri
IR 106523-3-9-3-2-B	$qDTY_{3.2}$ (RM231, RM517),<br>$qDTY_{12.1}$ (RM28048, RM511, RM28199, RM28166)	Savitri
IR 106531-13-31-2-3-B	$qDTY_{3.2}$ (RM231, RM517)<br>$qDTY_{12.1}$ (RM28048, RM511, RM28199, RM28166)	Savitri
IR 106529-15-33-1-2-B	$qDTY_{3.2}$ (RM231, RM517)<br>$qDTY_{12.1}$ (RM28048, RM511, RM28199, RM28166)	Savitri

Supplementary Table S2. List of NILs used in the present study for QTL interaction studies

Set of NILs	Recurrent parent	Donor parent	QTL and QTLs combinations	Number of low yielding NILs	Number of high yielding NILs	References
1	IR64	IR 86918-B-315, Way Rarem	$qDTY_{1.2}$ +$qDTY_{12.1}$	2	5	Swamy et al. 2013
2	IR64	Way Rarem	$qDTY_{12.1}$	4	5	Swamy et al. 2013
3	IR64	IR 86918-B-315, Way Rarem	$qDTY_{1.1}$ + $qDTY_{1.2}$ +$qDTY_{12.1}$	1	1	Swamy et al. 2013
4	IR64	IR 77298-14-1-2-17, Vandana	$qDTY_{2.2}$ + $qDTY_{2.3}$	1	2	Swamy et al. 2013
5	IR64	Vandana	$qDTY_{2.3}$ +$qDTY_{3.2}$	1	1	Swamy et al. 2013
6	IR64	Vandana	$qDTY_{2.3}$	2	2	Swamy et al. 2013
7	IR64	IR 77298-14-1-2-17	$qDTY_{4.1}$	1	1	Swamy et al. 2013
8	IR64	IR 77298-14-1-2-17, Way Rarem	$qDTY_{4.1}$ +$qDTY_{12.1}$	1	3	Swamy et al. 2013
9	TDK1-*Sub1*	IR55419–04	$qDTY_{6.1}$ + $qDTY_{6.2}$	2	1	Dixit et al. 2014; Dixit et al. 2017b
10	TDK1-*Sub1*	IR55419–04	$qDTY_{3.1}$ + $qDTY_{6.1}$+ $qDTY_{6.2}$	1	1	Dixit et al. 2014; Dixit et al. 2017b
11	Savitri	IR 77298-5-6-18	$qDTY_{3.2}$	1	1	Mishra et al. 2013; Yadaw et al. 2013; Dixit et al. 2017a
12	Savitri	IR 77298-5-6-18, IR74371-46-1-1	$qDTY_{3.2}$+ $qDTY_{12.1}$	2	2	Mishra et al. 2013; Yadaw et al. 2013; Dixit et al. 2017a

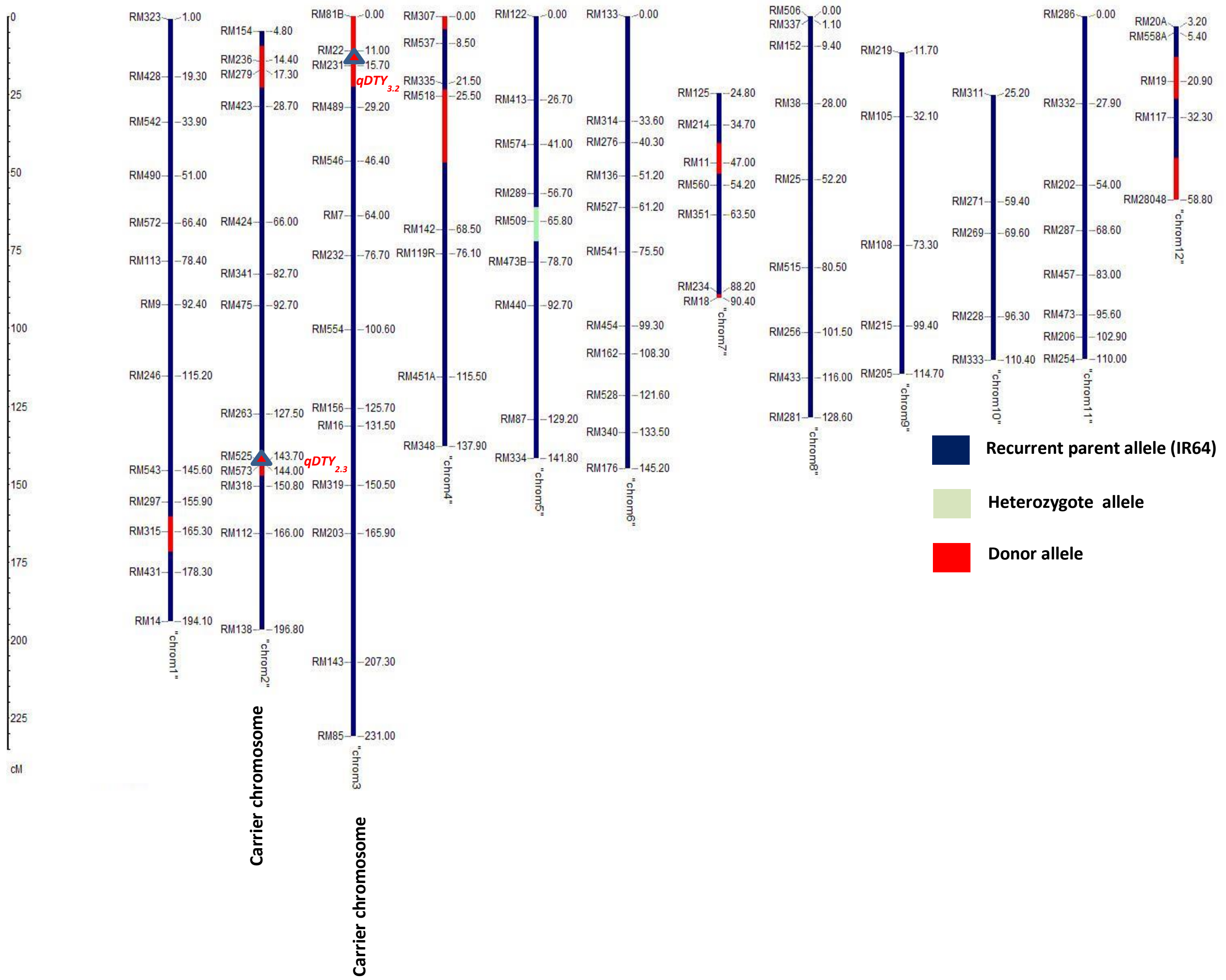

Supplementary Fig. S1 Graphical genotype representing the recurrent parent genome (RPG) recovery of IR64 NIL (IR 102784:2-42-16-1-1-2) carrying *$qDTY_{2.3}$*, *$qDTY_{3.2}$* drought QTLs on carrier chromosomes 2 and 3

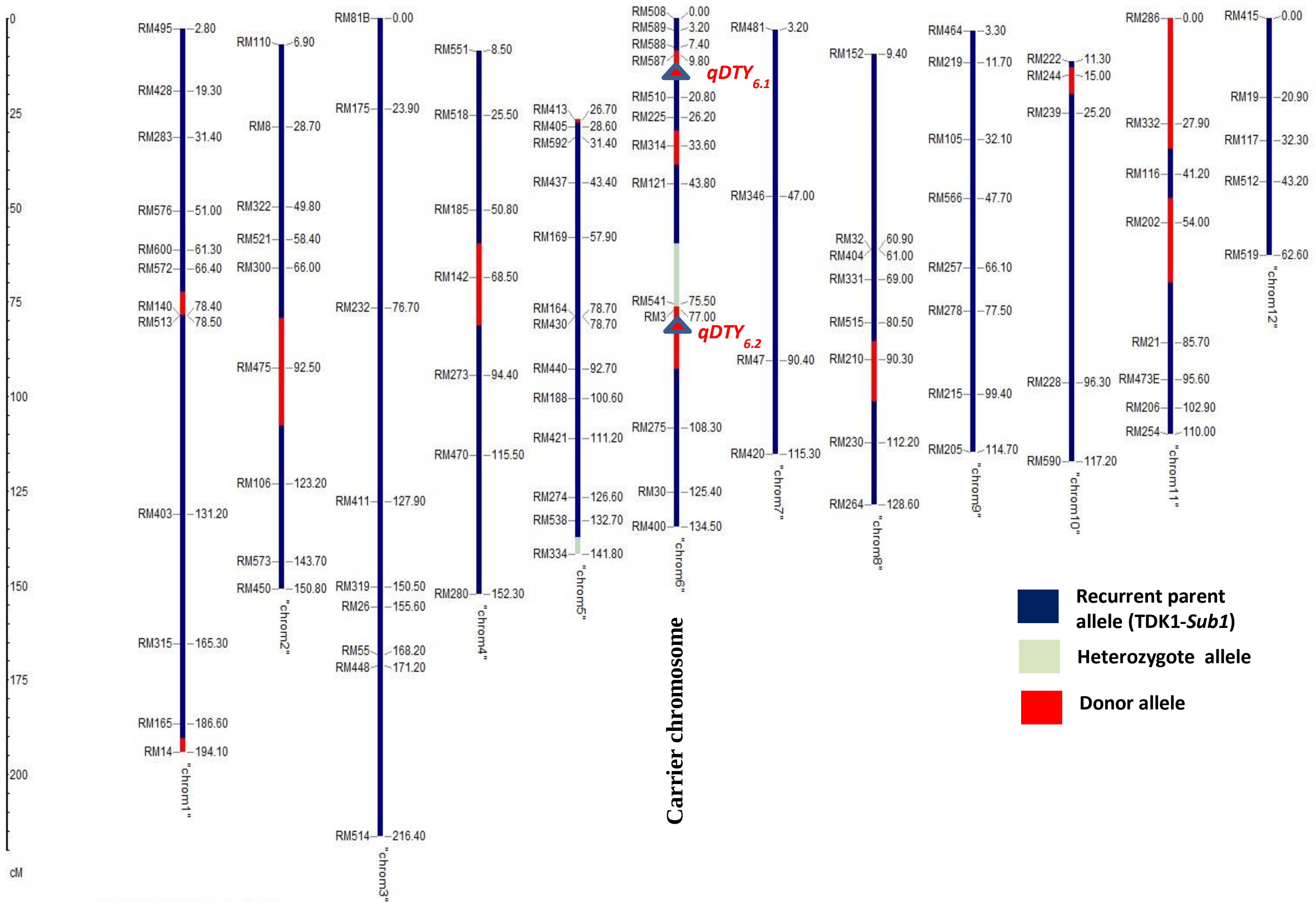

Supplementary Fig. S2 Graphical genotype representing the recurrent parent genome (RPG) recovery of TDK1-*Sub1* NIL (IR102777-5-83-127) carrying $qDTY_{6.1}$, $qDTY_{6.2}$ drought QTLs on carrier chromosome 6

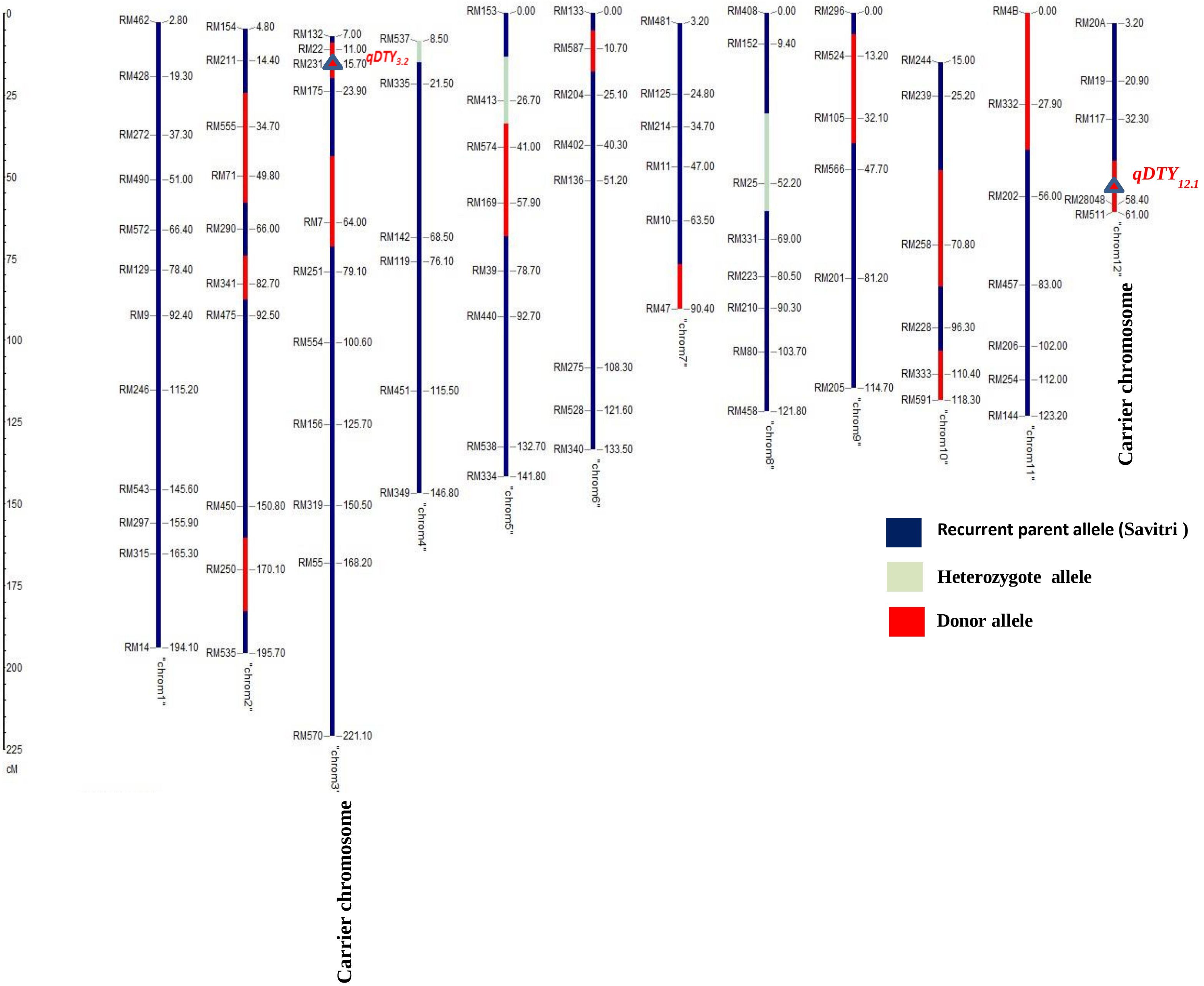

Supplementary Fig. S3 Graphical genotype representing the recurrent parent genome (RPG) recovery of Savitri NIL (IR 106523-21-28-1-2-B) carrying $qDTY_{3.2}$, $qDTY_{12.1}$ drought QTLs on carrier chromosomes 3 and 12

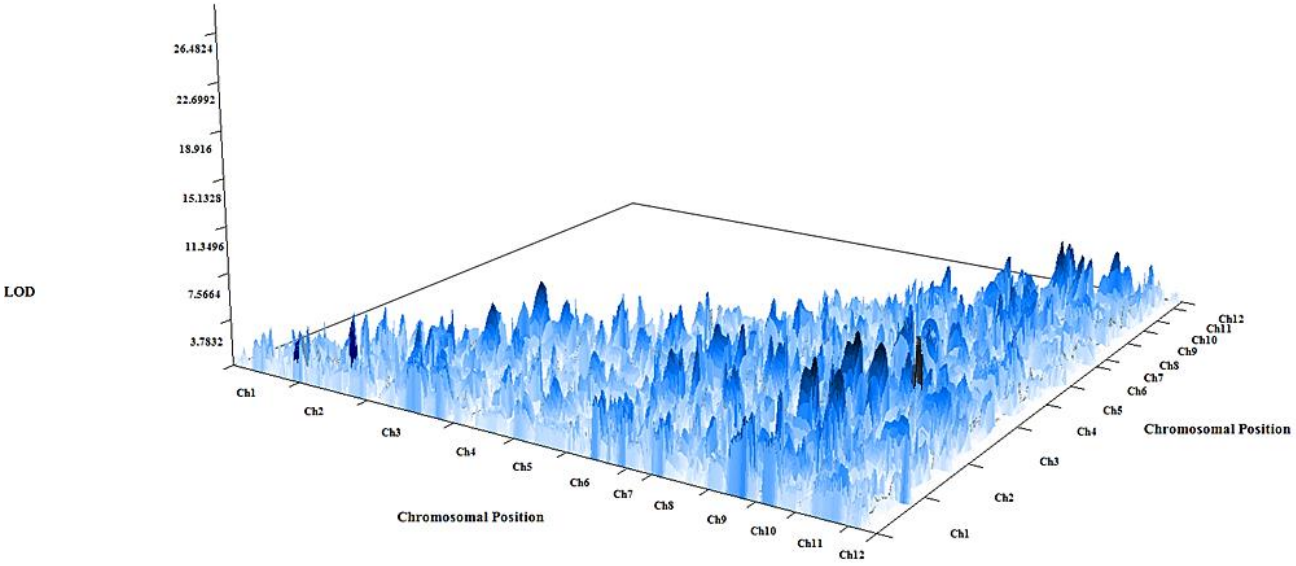

Supplementary Fig. S4 Heat map reflecting significant epistatic interactions according to LOD values among low yielding NILs of IR64 background under severe and moderate stress conditions

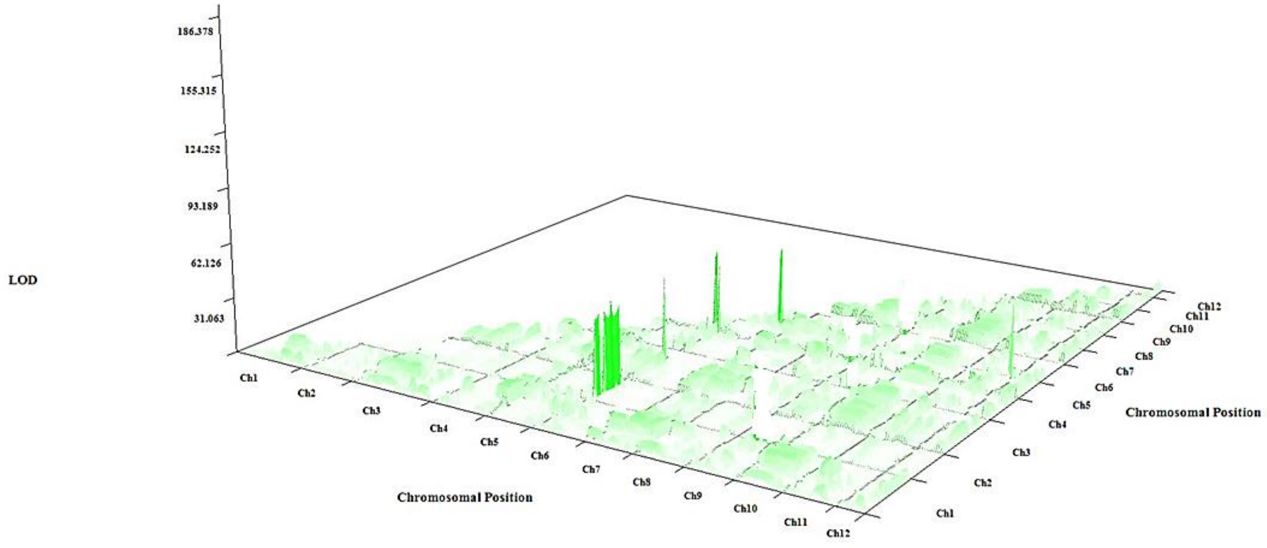

Supplementary Fig. S5 Heat map reflecting significant epistatic interactions according to LOD values among low yielding NILs of TDK1Sub1 background under severe stress conditions

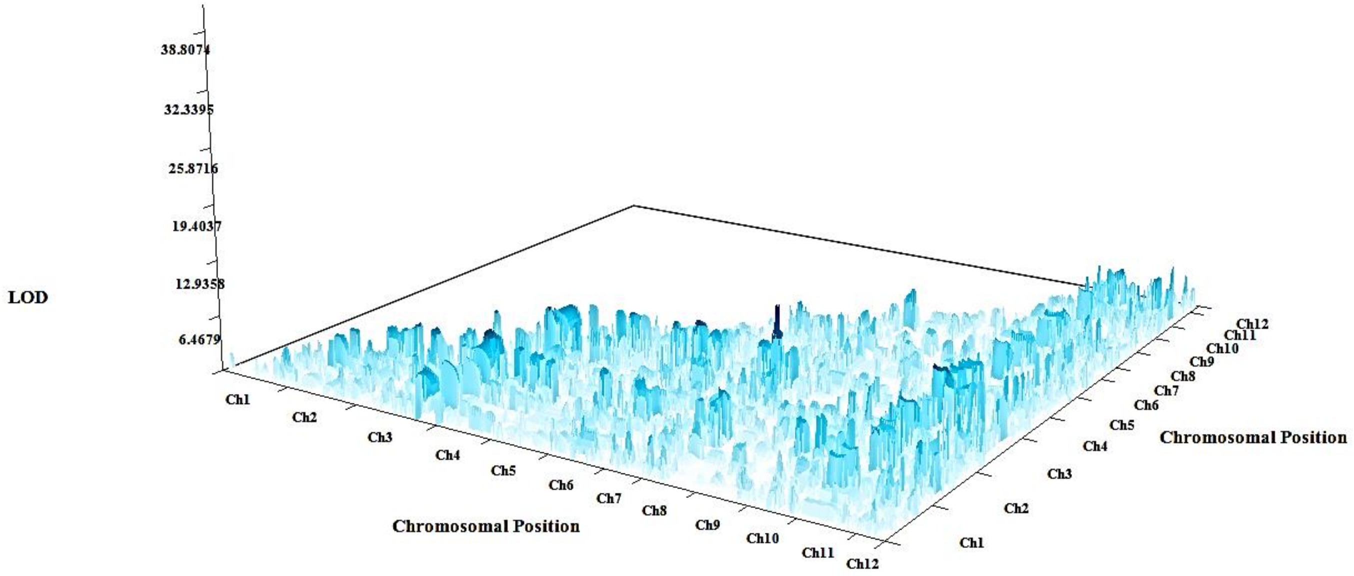

Supplementary Fig. S6 Heat map reflecting significant epistatic interactions according to LOD values among low yielding NILs of Savitri background under severe and moderate stress conditions
